# AEPMA: peptide–microbe association prediction based on autoevolutionary heterogeneous graph learning

**DOI:** 10.1093/bib/bbaf334

**Published:** 2025-07-10

**Authors:** Zhiyang Hu, Linqiang Pan, Daijun Zhang, Yannan Bin, Yansen Su

**Affiliations:** Key Laboratory of Image Information Processing and Intelligent Control, Ministry of Education, School of Artificial Intelligence and Automation, Huazhong University of Science and Technology, 430074 Wuhan, China; Key Laboratory of Intelligent Computing and Signal Processing, Ministry of Education, School of Artificial Intelligence, Anhui University, Jiulong Road, 230601 Hefei, China; Key Laboratory of Image Information Processing and Intelligent Control, Ministry of Education, School of Artificial Intelligence and Automation, Huazhong University of Science and Technology, 430074 Wuhan, China; Key Laboratory of Intelligent Computing and Signal Processing, Ministry of Education, School of Artificial Intelligence, Anhui University, Jiulong Road, 230601 Hefei, China; Key Laboratory of Intelligent Computing and Signal Processing, Ministry of Education, School of Artificial Intelligence, Anhui University, Jiulong Road, 230601 Hefei, China; Key Laboratory of Intelligent Computing and Signal Processing, Ministry of Education, School of Artificial Intelligence, Anhui University, Jiulong Road, 230601 Hefei, China

**Keywords:** antimicrobial peptide, microbe, heterogeneous network, autoevolutionary heterogeneous graph, graph convolutional networks

## Abstract

The inappropriate use of antibiotics has precipitated the emergence of multidrug-resistant bacteria, prompting significant interest in antimicrobial peptides (AMPs) as potential alternatives to traditional antibiotics. Given the prohibitive costs and time-consuming nature of biological experiments, computational methods provide an efficient alternative for the development of AMP-based drugs. However, existing computational studies primarily focus on identifying AMPs with antimicrobial activity, lacking a targeted identification of AMPs against specific microbial species. To address this gap, we propose a peptide–microbe association (PMA) prediction framework, termed AEPMA, which is constructed based on an autoevolutionary heterogeneous graph. Within AEPMA, we construct an innovative peptide-microbe-disease network (PMDHAN). Furthermore, we design an autoevolutionary information aggregation mechanism that facilitates the representation learning of the heterogeneous graph. This model automatically aggregates semantic information within the heterogeneous network while thoroughly accounting for the spatiotemporal dependencies and heterogeneous interactions in the PMDHAN. Experiments conducted on one peptide-microbe and three drug–microbe association datasets demonstrate that the performance of AEPMA outperforms five state-of-the-art methods, demonstrating its robust modeling capability and exceptional generalization ability. In addition, this study identifies a novel anti-Staphylococcus aureus peptide and an anti-Escherichia coli peptide, thereby contributing valuable information for the development of antimicrobial drugs and strategies for mitigating antibiotic resistance.

## Introduction

Microbes, including bacteria, fungi, and viruses, are tiny organisms that exist inside and outside the human body. Many diseases, whether infectious or chronic, may be closely related to microbial infections [1]. Furthermore, with the continuous emergence of drug-resistant microbes, many antibiotics that target these microbes have gradually lost their effectiveness. In the face of these pathogens, especially multidrug-resistant microbes, developing alternative therapies has become particularly important. Antimicrobial peptides (AMPs), as bio-molecules with therapeutic potential, exhibit broad-spectrum antimicrobial activity against various Gram-negative bacteria, cancer cells, and fungi [2]. Compared to traditional antibiotics, AMPs interact with microbial membranes and induce membrane permeation, effectively causing the death of the target microbes and reducing the development of resistance [3]. A deeper understanding of the complex interactions between AMPs and microbes can significantly enhance the selectivity and efficacy of treatments. Therefore, accurately predicting the associations between AMPs and microbes holds tremendous potential in the fields of AMPs research, development, and personalized medicine [4].

Currently, the study of AMPs primarily relies on biological experimental validation methods. However, these methods require a lot of human and material resources, and the long experimental period also limits the speed of the development of AMPs [5]. With the development of artificial intelligence technology, more and more computational methods are applied to the field of drug discovery and development. Recently, computational studies on AMPs mainly focus on utilizing sequence or structural features of AMPs to identify those with antimicrobial activity. For example, machine learning-based AMP classification methods such as CAMPr3 [6], iAMPpre [7], AmPEP [8], and CSAMPPred [9] use various sequence features (e.g. amino acid composition, pseudo amino acid composition, charge, and isoelectric point) as input features. These features are combined with classification methods like random forests and support vector machines to predict the antimicrobial activity of AMPs in a binary classification task. In addition to traditional machine learning methods, researchers have recently developed a series of deep learning-based approaches for identifying AMPs and their subfunctions (e.g. antibacterial, antiviral, and antifungal activities). Notable examples include methods such as iAMPCN [10], TransimbAMP [11], and iAMP-CA2L [12]. While these approaches have achieved some success, the vast diversity of microbes poses a challenge, as current AMP prediction studies are largely limited to the phylum and kingdom levels, lacking targeted AMP identification for specific microbial species. Advancing this research is critical for discovering hit and lead compounds in AMP development.

Research on drugs targeting specific microbes has focused on small molecule drugs, as reflected in microbe-small molecule drug association (MDA) prediction studies. For example, GCNMDA introduced conditional random fields into the graph convolutional network (GCN) framework to enhance the ability to predict associations between microbes and drugs [13]. Graph2MDA used variational graph autoencoders (VGAEs) to extract latent representations of microbes and drugs, thereby achieving the prediction of microbe–drug associations (MDAs) [14]. SCSMDA guided GCNs with meta-paths to learn node features and combined contrastive learning to enhance the features extracted from microbe and drug similarity networks [15]. In addition, the method used a negative sampling strategy to optimize the training of multilayer perceptron classifiers to predict potential MADs. The success of the existing microbe-specific targeted drug studies demonstrates the technical feasibility of studying microbe-specific AMPs, and methodological ideas can be borrowed to guide the research related to AMPs.

With the development of graph neural network (GNN), GNN models have demonstrated their efficacy in capturing the intricate topological structure of heterogeneous networks. Tabei *et al*. constructs a multi-view heterogeneous network, updates node features through neighborhood information aggregation layers, and predicts associations between biological entities using a discriminator after integrating the combined embeddings [16]. However, this method still lacks sufficient interpretability and provides limited consideration of the complex relationships between biological entities. Recently, meta-path-based prediction models have become an effective strategy to identify neighboring nodes in a heterogeneous network and thus aggregate node and edge information in heterogeneous networks [17]. Specifically, meta-paths in biologically heterogeneous networks may include specific metabolic pathways or biological principles, which facilitate the interpretation of PMA predictions [18]. For example, Tanvir et al. [19] employed meta-paths to extract rich semantic relationships between entities and fed the integrated features into a classifier for prediction of drug-drug interaction. These meta-paths are always empirically designed, heavily dependent on domain knowledge, and challenging to transfer to other heterogeneous networks. Furthermore, although heterogeneous networks include a wide variety of information, not all are useful for prediction. Recent advances in network structure search techniques have shown the effectiveness of developing an adaptive network structure for PMA prediction. HampDTI designed a trainable meta-path based on network structure search techniques and used the generated meta-path graph to learn low-dimensional features of drugs and targets [20]. Compared to trainable meta-paths, meta-graphs have a higher order ability to capture complex semantic information [21].

To address these issues, we propose a novel peptide–microbe association PMA prediction method based on an autoevolutionary heterogeneous graph. First, a heterogeneous network comprising peptides, microbes, and diseases is constructed, where associations between peptides and microbes and between microbes and diseases are obtained from the DBSSP [22] and Disbiome databases [23]. The similarity between peptides is calculated using the Smith-Waterman algorithm, while the similarity between microbes and diseases is obtained through Gaussian kernel interaction profiles. Then, based on the similarities among peptides, microbes, and diseases, initial features for peptides, microbes, and diseases are obtained using a random walk with restart (RWR) algorithm. Finally, the association scores between peptides and microbes are obtained by computing the inner product of peptide and microbe features, thus predicting the potential peptides for different microbes. This model aims to facilitate the discovery of peptides and improve the efficiency of peptides development. Specifically, the main contributions of this paper are summarized as follows.

(1) We constructed a heterogeneous network to efficiently integrate diverse biological data, including similarity information on microbes, peptides, and diseases, as well as associations between microbes and diseases, and between peptides and microbes.
(2) We propose an autoevolutionary heterogeneous graph search strategy that automatically searches for an efficient way to integrate information without relying on domain knowledge. The autoevolutionary heterogeneous graph has a more flexible structure to efficiently represent fine-grained complex semantic information and is used to learn the complex topology of heterogeneous networks to infer potential relationships between peptides and microbes.

## Materials and methods

**Figure 1:**
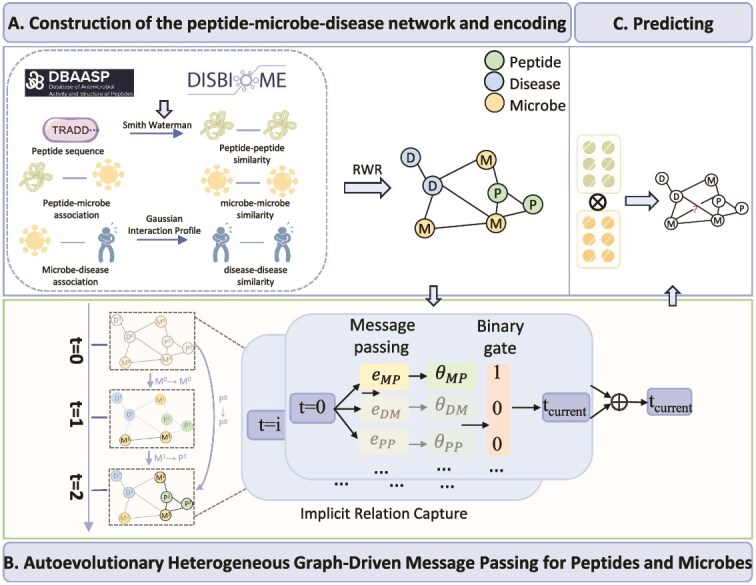
Overview of the AEPMA algorithm, which is divided into three steps. (A) Constructing the PMDHAN, with multi-source biomedical data and employing the RWR algorithm to encode the node representation. (B) Autoevolutionary message passing for peptides and microbes. (C) Utilizing the inner product of the aggregated feature representation of peptides and microbes to predict potential PMA.

In this work, we propose a novel framework AEPMAs based on **A**uto**E**volutionary heterogeneous graph to predict PMAs. The framework of AEPMA is shown in Fig. 1. First, we specifically construct a new peptide-microbe-disease network PMDHAN, and learn the initial features of peptides, microbes, and diseases by employing the RWR method on the network. Then, we design an autoevolutionary heterogeneous graph to comprehensively consider the spatiotemporal dependencies and heterogeneous interactions within the PMDHAN. Finally, the features of peptides and microbes are multiplied to obtain PMA scores, thereby discovering potential PMAs. Next, we will detail the steps mentioned above.

### Construction of the peptide-microbe-disease network

In our work, we collected the PMAs and microbe-disease associations from two public databases, and calculated the similarity among peptides, microbes and diseases. Specifically, the PMAs are collected from the database of antimicrobial activity and structure of peptides (DBAASPs), since DBAASP curated abundant PMAs as well as the sequences of $22\,257$ peptides, and is a powerful tool to understand the functions of peptides [22]. To minimize bias from overly similar or repetitive peptides and ensure diversity in sequence features, we selected peptides based on the length and redundancy of peptides. Specifically, we filtered peptides with length larger than $50$ amino acids, and then removed redundant sequences using the CD-HIT tool with $0.7$ as the threshold. Finally, we achieved associations between 4050 peptides and microbes. In addition, we retrieved $10\,866$ associations between $1615$ microbes and $375$ diseases from the Disbiome database. The Disbiome database standardizes and organizes the associations between microbes and diseases, which helps researchers analyze complex relationships and provides support for exploring disease mechanisms and developing therapeutic interventions [23]. Due to the inconsistencies in microbes between DBAASP and Disbiome, we selected the microbes with associations in both DBAASP and Disbiome. In addition, we adopted the binomial nomenclature system to normalized the name of microbes. In this way, peptides and diseases can be associated with the same set of microbes. Finally, we achieved $670$ microbe–disease associations and $16\,205$ PMAs, where the adjacency matrices $A_{PM}$ and $A_{MD}$ respectively denotes PMAs, and microbe–disease associations. Besides, we also applied the Smith-Waterman [24] algorithm to calculate the similarity between peptides based on their sequences, and used the Gaussian Interaction Profile [25] to calculate the similarity between microbes and diseases based on the microbe–disease association matrix. More details about the similarity calculations can be found in Supplementary Materials. The PMDHAN contains the PMAs, the microbe-disease associations, as well as the peptide-peptide similarities, microbe-microbe similarities, and disease-disease similarities. The details of the PMDHAN are listed in Table 1.

**Table 1:** The details of the PMDHAN

Relationship type	Peptides	Microbes	Diseases	Associations
Peptide-microbe	4050	131	-	16 205
Microbe-disease	-	131	161	670
Peptide-peptide	4050	-	-	16 402 500
Microbe-microbe	-	131	-	17 161
Disease-disease	-	-	161	25 921

### Encoding for peptides, microbes, and diseases

In our study, we use RWR to extract the initial features of peptides, microbes, and diseases from the similarity networks of peptides, microbes, and diseases, since RWR is good at capturing both local and global topological features of the network and has been widely applied in denoising in image processing and in preserving proximity information in feature learning [26]. In this way, we obtain probability distribution vectors for peptides, microbes, and diseases. To ensure comparability of features across different nodes, we further normalized the values in probability distribution vectors. Here, the normalized probability distribution vectors are used as the initial features for peptides, microbes, and diseases.

### Autoevolutionary message passing for peptides and microbes

Traditionally, meta-paths and meta-graphs describe modes of message passing. However, these meta-paths/graphs-based message passing modes are always fixed and rely on domain knowledge in the manual design situation, which may limit the flexibility of information transmission and prevent it from fully adapting to the complex relationships in the task. In this section, we proposed an autoevolution representation for peptides and microbes, where the representation for peptides and microbes at a timestamp is achieved by contacting those achieved before the timestamp, and the message passing modes at each timestamp is automatically selected. It is worth noting that the autoevolutionary representation for peptides and microbes is described as the features of nodes in an autoevolutionary heterogeneous graph. In what follows, the definition of an autoevolutionary heterogeneous graph and representation learning of an autoevolutionary heterogeneous graph are illustrated.

#### Definition of an autoevolutionary heterogeneous graph

Here, an autoevolutionary heterogeneous graph is comprised of multiple embeddings of a given heterogeneous network (e.g. the PMDHAN) at different timestamps. Specifically, the feature of the given heterogeneous network at the timestamp $t$ is denoted as $G^{t}=<N^{t},E^{t}>$, where $N^{t}$ represents the features of all nodes in the heterogeneous network at the current timestamp $t$, which are obtained from the features at the timestamp $i$ before $t$ (i.e. $i \in \{0,\dots ,t-1\}$) through a certain information propagation mode $e^{i,t}$. $E^{t}=\{e^{i,t}|i=0,\dots ,t-1\}$ is the set of message passing modes, where $e^{i,t}$ guides the message passing of the heterogeneous network from the timestamp $i^{th}$ to the timestamp $t^{th}$. It is noted that each information aggregation mode $e^{i,j}$ is automatically selected from all possible information aggregation modes in the heterogeneous network. For example, in the PMDHAN, the possible information aggregation modes includes the “microbe$\rightarrow $ peptide,” the “peptide$\rightarrow $ microbe,” “microbe$\rightarrow $ disease,” “disease$\rightarrow $ microbe,” “microbe$\rightarrow $ microbe,” “peptide$\rightarrow $ peptide,” and “disease$\rightarrow $ disease” in terms of the edge types. Besides, the features at the timestamp $i$ may not affect those in current timestamp $j$. Further, the features at the timestamp $j$ may also inherit the features at the timestamp $i$. Thus, all of the possible information aggregation modes for the PMDHAN can be formed as the set $A=\{ e_{MP}, e_{PM}, e_{MD}, e_{DM}, e_{MM}, e_{PP}, e_{DD}, e_{\emptyset }, e_{I} \}$, where $e_{XY}$ represents the information aggregation mode “X$\rightarrow $ Y,” $X$ and $Y\in \{microbe (M), peptide(P), disease(D)\}$; $A_\emptyset $ denotes that a previous timestamp could not affect the current timestamp; $A_{I}$ means that the features at the current timestamp inherit the features at a previous timestamp. Specially, the embedding of the PMDHAN in the initial time is $G^{0}=<N^{0}, E^{0}>$. In this situation, $N^{0}$ is the initial node features constructed using RWR and $E^{0}=\emptyset $, since there is no message passing in the initial state. If the information aggregation mode from the timestamp $0$ to $1$ is labeled as “microbe$\rightarrow $microbe”(Fig. 1B), it indicates that the features at the timestamp $1$ are achieved by aggregating the features of the “microbe” nodes to the features of the “peptide” nodes in the network at the timestamp $0$.

An autoevolutionary heterogeneous graph is formally defined as follows: 


(1) \begin{align*} \mathcal{G} = \left\{\{G^{t}\}_{t=0}^{T} \right\},\end{align*}

where $T$ is the final timestamp. In this graph, any of the feature in the previous timestamps can influence those at the current timestamp through a certain information propagation mode, thereby forming a jump structure between different timestamps in the autoevolutionary heterogeneous graph. Therefore, a prominent feature of the autoevolutionary heterogeneous graph is the jump structure between timestamps, which enables it to extract complex semantic information more effectively from a given heterogeneous network.

#### Representation learning of an autoevolutionary heterogeneous graph

**Figure 2:**
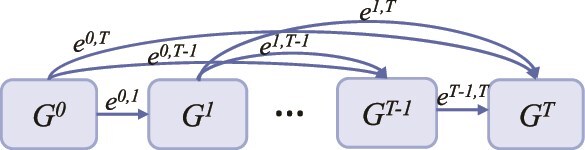
Schematic diagram of representation learning in autoevolutionary heterogeneous graph.

Representation learning of nodes in heterogeneous graphs is an “evolutionary” process. The initial features of the nodes at the starting timestamp are obtained based on an initialized representation learning method. For instance, in the PMDHAN, the initial features of nodes are derived from RWR. Given that the features corresponding to timestamps prior to $t$ have been learned, the features at the current timestamp $t$ are prone to be affected by those at the timestamp before $t$, where the features at the timestamp $i$ can be propagated into those at the timestamp $t$ by a possible aggregation mode with a certain probability. To make the feature representation of this heterogeneous graph suitable for the task of predicting relationships between peptides and microbes, the crucial step in learning the representation of a heterogeneous graph from $i$ to $t$ lies in the selection of the information aggregation mode from $i$ to $t$. In what follows, we first elaborate on the methodology for aggregating information from the timestamp $i$ to $t$ based on the given information aggregation modes. Then, we give the details for identifying the most suitable information aggregation mode for a particular prediction task. The schematic diagram of representation learning in autoevolutionary heterogeneous graph is shown in Fig. 2.

Here, given a timestamp $i$ i.e. prior to the timestamp $t$, we assume that the features at timestamp $i$ influence the features at timestamp $t$ in the form of the information aggregation mode $e$ with a probability of $\theta _{e}^{i,t}$. Then, the features at the timestamp $t$ influenced by those at the timestamp $i$, denoted as $N^{i,t}$, are calculated based on GCN model by Equation (2). 


(2) \begin{align*} N^{i,t} = \sum_{e \in A} {\theta}^{i,t}_{e} \cdot \left(D^{-\frac{1}{2}} e D^{-\frac{1}{2}} N^{i}\right),\end{align*}

where $D$ is the degree matrix of the nodes, $N^{i}$ is the node feature at the timestamp $i$, and $A$ denotes the set of possible aggregation modes. It is noted that the possible aggregation modes in $A$ are related with the prediction task as well as the timestamp. For example, for the prediction task of PMAs, if $t \ne T$ ($T$ is the final timestamp), then the influence pattern from $i$ to $t$ could be any one of the possible information aggregation modes in $A=\{ e_{MP}, e_{PM}, e_{MD}, e_{DM}, e_{MM}, e_{PP}, e_{DD}, e_{\emptyset }, e_{I} \}$. In the PMAs prediction task, to achieve the embeddings of peptides and microbes at the last timestamp, we have imposed constraints on the information aggregation modes. These constraints are designed to focus on the patterns where peptides and microbes serve as endpoints. By doing so, we ensure that the model selectively captures the most relevant patterns and features, effectively filtering out unnecessary or irrelevant information. Thus, if $t = T$, then the possible aggregation modes are in the form of “$*\rightarrow $peptide” or “$*\rightarrow $microbe,” i.e. $A=\{ e_{MP}, e_{PM}, e_{DM}, e_{MM}, e_{PP} \}$.

Further, the features at the timestamp $t$ is the cumulative summation of those influenced from all preceding timestamps, which is calculated as Equation (3). 


(3) \begin{align*} N^{t} = \text{GELU} \left( \sum_{i = 0}^{t-1}N^{i,t} \right),\end{align*}

where $N^{i,t}$ is the feature influenced from the timestamp $i$, and GELU is the activation function.

To determine the optimal information propagation mode for PMAs, it is necessary to simultaneously optimize the model’s conventional parameters (e.g. hidden weight matrices and biases) and the probability parameters of the information propagation patterns. Let $\omega $ denotes the conventional parameters of the proposed model, and $\Theta = \left \{ \theta _{e_{1}}^{0,1}, \cdots ,\theta _{e_{m}}^{0,2},\cdots ,\theta _{e_{m}}^{t-1,t}\right \}$ denote the set of all probability parameters of information aggregation modes. The selection of the most suitable information aggregation mode is modeled as a dual-layer optimization framework, where one layer optimizes the probability parameters $\Theta $ by minimizing the validation loss $\mathcal{L}_{val}$ and the other layer optimizes the model parameters $\omega $ by minimizing the training loss $\mathcal{L}_{tra}$, which is calculated as Equation (4) and Equation (5). 


(4) \begin{align*} \min_{\Theta} \operatorname{\mathcal{L}_{\text{val}}} (\boldsymbol{\omega}^{*}(\Theta), \Theta), \text{ s.t. } \boldsymbol{\omega}^{*}(\Theta)=\arg \min_{\boldsymbol{\omega}} {\mathcal{L}}_{\text{tra }}(\boldsymbol{\omega}, \Theta), \end{align*}

(5) \begin{align*} \mathcal{L}=-\sum_{(p, m) \in \Omega^{+}}\log \sigma\left(N_{p} \cdot N_{m}\right)-\sum_{\left(p^{\prime}, m^{\prime}\right) \in \Omega^{-}} \log \sigma\left(-N_{\mathrm{p}^{\prime}} \cdot N_{m^{\prime}}\right)\!, \end{align*}

where $\Omega ^{+} $ is the set of known PMAs (positive samples), and $\Omega ^{-} $ is the negative samples that contain the peptide and microbe without associations. $N_{p}$, $N_{m}$ are the node representations of peptides and microbes obtained from positive samples, while $N_{p^{\prime}}$, $N_{m^{\prime}}$ are those achieved from negative samples, respectively. $\log \sigma $ denotes the logsigmoid function.

### PMA prediction

Upon completion of the information aggregation process, we derived the feature representations for both peptides and microbes. To calculate the prediction score for peptide-microbe node pairs, we utilized the inner product of these two feature vectors. The inner product operation can effectively capture the degree of alignment between the peptide and microbe in the feature space. Mathematically, the inner product reflects both the directional similarity and the magnitude relationship between two vectors: a larger inner product value indicates a closer proximity between the representations of the peptide and the microbe, suggesting a stronger potential for binding between them. For a given peptide $p$ and a specific microbe $m$, the association score $\mathcal{P}^{pm} $ between $p$ and $m$ can be calculated as Equation (6). Subsequently, the resulting inner product score is normalized by a sigmoid function, which maps the score to the interval $[0, 1]$, thereby representing the probability of association between the peptide and the microbe. 


(6) \begin{align*}& \mathcal{P}^{p m}=sigmoid\left(N_{p} \cdot N_{m}\right),\end{align*}

where $N_{p} $ and $N_{m} $ are the feature representations of peptides and microbes, respectively.

## Results

In this section, we verified the performance of the proposed AEPMA by comparing it with five methods.

### Baseline methods

To the best of our knowledge, there is a lack of methods specifically tailored to solve the problem of predicting PMAs. In our work, the proposed AEPMA model is compared with five state-of-the-art neural network-based methods proposed for drug–microbe association prediction, namely GCNMDA, MMGCN, MKGCN, Graph2MDA, and SCSMDA.

- GCNMDA [13] learns the low-dimensional node representations from heterogeneous networks based on a GCN and a conditional random field layer with an attention mechanism for the predictions of drug–microbe associations.
- MMGCN [27] employs GCN to compute the representations of microbes and drugs from different similarity perspectives. Then, a multi-channel attention mechanism adaptively adjusts these features to determine the potential response of microbes to drugs.
- MKGCN [28] employs GCN to extract multi-layer features and performs association prediction in microbe-drug space by computing kernel matrices layer by layer and weighted fusion combined with pairwise Laplace regularized least squares.
- Graph2MDA [14] is an integrated framework combining the VGAE and deep neural networks (DNNs). VGAE is used to learn the attributes of drugs and microbes, as well as the information from heterogeneous networks, while the DNN classifier predicts the association probabilities between drugs and microbes.
- SCSMDA [29] learns the embeddings of drugs and microbes by a specially designed meta-path, and further enhances feature learning by using a contrastive learning strategy.

### Experiment settings and evaluation measures

The proposed AEPMA model is implemented on the PyTorch framework with the Adam optimizer [30], where the learning rate is set to $6 \mathrm{e}{-3}$, the weight decay rate to $1 \mathrm{e}{-3}$, the hidden size to $64$, the decay rate to $0.2$ and the epoch to $100$. For RWR, the maximum number of steps for random walks is set to $80$, the number of walks is $10$, and the probability of restart is $0.2$. Moreover, we use two well-known performance metrics to assess the performance of model, namely the Area Under the Receiver Operating Characteristic Curve (AUC) and the Area Under the Precision-Recall Curve (AUPRC).

### Performance evaluation

To evaluate the performance of the AEPMA model, we conduct experiments using the default model parameters of the comparison methods, adjust the hyperparameters for deep learning in these competing methods, and perform five-fold cross-validation on the PMDHAN dataset. Specifically, a randomly chosen subsets of $60\%$ and $20\%$ of the known PMAs and a matching number of non-interacting pairs are respectively held out as the training set and the validation set, and the remaining $20\%$ of the PMAs and a matching number of non-interacting pairs are used to test the model. In our work, five runs of five-fold cross-validation are performed on the PMDHAN dataset. Table 2 shows the average values of AUC and AUPRC achieved by each model. From Table 2, the following observations can be made.

**Table 2:** Comparison of AUC and AUPRC values between different models

Method	PMDHAN	
	AUC	AUPRC
GCNMDA	0.954$\,\pm\, $0.0017	0.956$\,\pm\, $0.0036
MMGCN	0.942$\,\pm\, $0.0042	0.943$\,\pm\, $0.0053
MKGCN	0.913$\,\pm\, $0.0032	0.923$\,\pm\, $0.0042
Graph2MDA	0.965$\,\pm\, $0.0072	0.964$\,\pm\, $0.0062
SCSMDA	0.952$\,\pm\, $0.0052	0.948$\,\pm\, $0.0049
AEPMA	**0.989$\,\pm\, $0.0018**	**0.989$\,\pm\, $0.0038**

Bold text in the table indicates the highest value in each column.

First, AEPMA consistently outperformed the other five methods, with AUC and AUPRC improvements of $2.4\%$ and $2.5\%$, respectively, over Graph2MDA, the model with the second highest performance. It is presumably because Graph2MDA learns low-dimensional node representations from heterogeneous networks based on a VGAE and DNN classifier, which fails to distinguish the information aggregation modes at different times. However, the proposed AEPMA applies the autoevolutionary heterogeneous graph module, which effectively captures complex associations in biological networks through dynamic graph structure learning and spatiotemporal dependency modeling.

Second, the proposed representation learning of an autoevolutionary heterogeneous graph is more suitable to learn node information than that based on meta-paths for the prediction of drug–microbe associations. Specifically, the AUC obtained by AEPMA is $0.989$, which is $3.7\%$ higher than that obtained by SCSMDA. That is presumably because the meta-path-based SCSMDA learns the embeddings of nodes in terms of the manually designed meta-path, which largely depends on the subjective consciousness of researchers. In contrast, the proposed representation learning of an autoevolutionary heterogeneous graph enables the automatic selection of appropriate information aggregation modes.

### Analysis of association sensitivity

**Figure 3:**
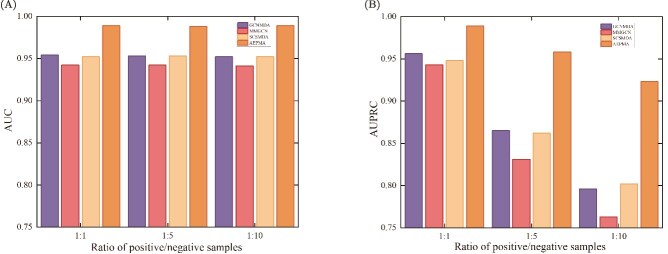
The performance of models with different positive and negative ratios on PMDHAN’s dataset. (A) Comparison of AUC values for models. (B) Comparison of AUPRC values for models.

To simulate a practical situation in which the peptide-microbe pairs are often labeled with only a few known PMAs, we also performed two additional five-fold cross-validation tests, in which the negative samples (unknown PMAs) in the training (validation, test) set are five times or ten times more than the positive ones (known PMAs). Figure 3A shows the AUC and AUPRC values of AEPMA when the ratios of positive to negative samples are $1:1$, $1:5$, and $1:10$. It is worth noting that since MKGCN and Graph2MDA adopt an unbalanced sample training strategy, their training paradigms fundamentally differ from the settings of this study, and thus they are not included in the comparative analysis. As can be observed from the figure, the AUC value remains relatively stable across different positive-to-negative sample ratios. However, the values of AUPRC achieved by all the models are decrease when compared to the previous test ($1:1$), and AEPMA still obtained much higher AUPRC than other compared models. As indicated in previous studies, the AUPRC can offer a more accurate assessment than the AUC when dealing with highly imbalanced data, since AUC tends to be an overly optimistic metric for evaluating the performance of a prediction algorithm in this situation. The above results indicate that the proposed method demonstrates a remarkably robust performance, scarcely affected by the ratio of known PMAs.

**Figure 4:**
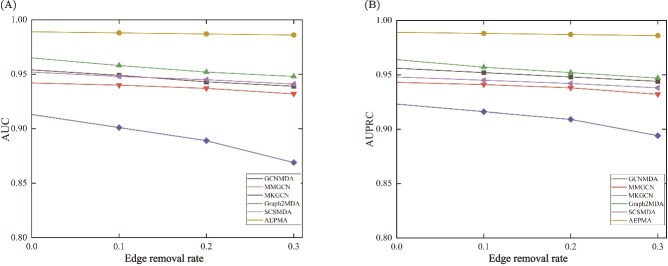
Comparison of the effect of edge deletion ratio on model performance on PMDHAN’s dataset. (A) Comparison of AUC values for models after removing edges. (B) Comparison of AUPRC values for models after removing edges.

The network-based PMA prediction methods may be influenced by the incompleteness of heterogeneous networks. In order to measure the association sensitivity of the proposed AEPMA, we randomly removed four proportions of associations within the heterogeneous network. Specifically, existing $0\%$, $10\%$, $20\%$, and $30\%$ of associations were randomly deleted and five-fold cross-validations were conducted for five times to evaluate the performance of AEPMA and other five compared methods. As shown in Fig. 4, the AUC and AUPRC values achieved by all the models decrease with the increase of the proportion of the deleted associations, but the proposed AEPMA still achieved the highest AUC and AUPRC values in each situation. It is indicated that AEPMA has a strong robustness in dealing with network information loss and can effectively handle situations where some information is missing in PMDHAN.

### Analysis of the autoevolutionary message passing mechanism

**Figure 5:**
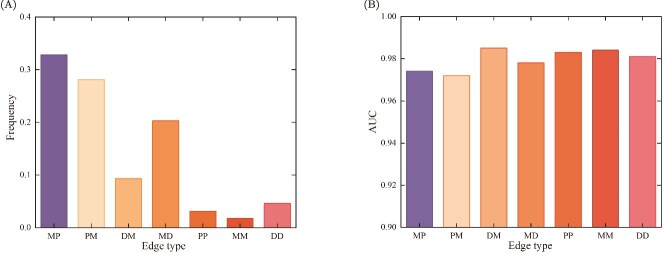
Comparison of edge type frequencies and AUC values. (A) Frequency statistics of different edge relationships. (B) Comparison of AUC values after excluded different edge relationships.

The information aggregation modes for the representation learning of an autoevolutionary heterogeneous graph are also a key mechanism of a GNN model. To explore the optimal information aggregation mode for the prediction of PMAs, additional five-fold cross-validations were conducted ten times on the PMDHAN dataset, resulting in ten autoevolutionary heterogeneous graphs. The frequency of edges in the ten autoevolutionary heterogeneous graphs was calculated. The frequency of each edge type is shown in Fig. 5A. As can be seen from the Fig. 5A, the ‘microbe $\rightarrow $ peptide’ association contributes the most to the prediction of PMAs, followed by the ‘peptide $\rightarrow $ microbe’ association.

To further investigate the contribution differences of different edge types to the prediction of PMAs, we evaluated the performance of the proposed AEPMA when removing each edge type from the original heterogeneous graph. Specifically, we sequentially removed specific types (e.g. ‘microbe-peptide’, ‘disease-microbe’, etc.) of associations in the PMDHAN, and applied a five-fold cross-validation strategy with five repetitions to quantify the fluctuation of model prediction performance, with AUC as the performance evaluation metric. The additional evaluation metrics are provided in Supplementary Material S3. Figure 5B shows the AUC values after removing each edge type from the original heterogeneous graph. As shown in Fig. 5B, the removal of different edge types leads to differential performance degradation. Specifically, the removal of the ‘microbe-peptide’ bidirectional interaction edge causes a significant $2\%$ decrease in AUC, while the removal of other edge types only results in a slight decrease of $1.3\%$–$1.9\%$. This correlation trend is highly consistent with the frequency distribution of edge types shown in Fig. 5A. It is indicated that AEPMA can effectively interpret key interaction patterns in biological networks.

**Figure 6:**
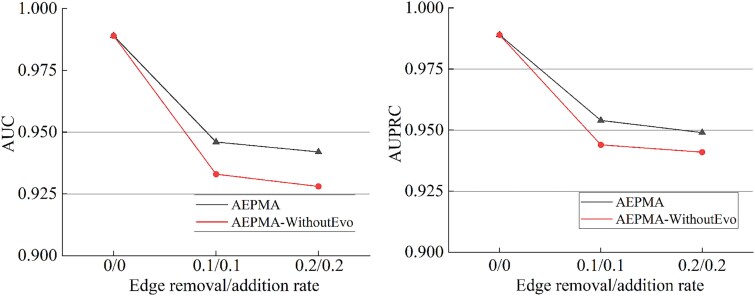
The performance of AEPMA with edge modification effects on PMDHAN’s dataset. AEPMA-WithoutEvo employs the original information aggregation mode.

To comprehensively evaluate the effectiveness of the auto-evolutionary information aggregation mode, we compared the performance of the original information aggregation mode (with fixed propagation paths) and the new information aggregation mode (with autoevolutionary propagation paths) on the original peptide–microbe–disease network and its structurally perturbed versions, where real edges were removed and noisy edges were added at ratios of $0\%/0\%$, $10\%/10\%$, and $20\%/20\%$, respectively. The evaluation focuses on the changes in AUC and AUPRC under different perturbation levels. As shown in Fig. 6, as network edges are removed and noisy edges are added, the performance of the original-information aggregation mode significantly declines, whereas each of the new-information aggregation modes, leveraging its adaptive mechanism, maintains a higher level of performance stability under structural perturbations. Furthermore, the new-information aggregation mode improves AUC and AUPRC by approximately $1.3\%$ compared to the original-information aggregation mode. These results indicate that in various complex scenarios, the new information aggregation mode demonstrates greater robustness and adaptability, dynamically adjusting its structure to accommodate network changes, thereby significantly enhancing the model’s prediction accuracy and stability.

### Ablation study

The proposed AEPMA mainly contains three essential components, i.e. encoding the nodes in a heterogeneous network by RWR, and autoevolutionary message passing for peptides and microbes, where the strategy of automatically selecting possible information aggregation mode is the key for autoevolutionary message passing. Here, the ablation experiments are conducted to assess the contribution of these key components. The model variants are summarized as follows: (i) AEPMA-RWR refers to AEPMA without RWR but with onehot encoding for initial node encoding; (ii) AEPMA-Constraints removes constrained link selection at the final timestamp when $t=T$; and (iii) AEPMA-All neighbors without autoevolutionary message passing but passing all the features of neighbors.

Compared to AEPMA, AEPMA-RWR exhibits a performance decline of approximately $1.4\%$, underscoring the crucial role of encoding nodes with RWR in enhancing PMA prediction accuracy. Similarly, AEPMA-Constraints experiences a performance drop of nearly $3\%$, emphasizing the benefits of incorporating task-relevant relationship types in the final stage of information transmission. Furthermore, AEPMA-All Neighbors shows a performance decrease of around $3.3\%$, reinforcing the effectiveness of integrating an autoevolutionary message-passing strategy in information aggregation. These comparisons systematically highlight the contribution of each component to the overall performance of the complete mode.

**Table 3:** Comparison of AUC and AUPRC values between different models

Method	PMDHAN	
	AUC	AUPRC
AEPMA-RWR	0.975	0.972
AEPMA-constraints	0.959	0.955
AEPMA-all neighbors	0.956	0.952
AEPMA	**0.989**	**0.989**

Bold text in the table indicates the highest value in each column.

### Cold start analysis

Existing studies have shown that graph neural networks can significantly improve prediction performance. However, when there is limited associated information for peptide or microbe nodes, the learning capability of most models will decrease substantially. This is a common cold-start scenario in real-world applications. To evaluate the model’s performance in cold-start scenarios, we conducted a cold-start analysis on the PMDHAN dataset, and the comparison results are shown in Table 4. Specifically, we first grouped ’cold’ peptides and microbe based on the quantity of associations and designed different degrees of cold start testing scenarios. In all testing scenarios, we maintained a $1:1$ ratio of positive to negative samples and used AUC and AUPRC as model performance metrics. It is worth noting that since Graph2MDA adopts an unbalanced sample training strategy, its training paradigm has fundamental differences from the setting of this study, and thus it was not included in the comparative analysis. As shown in Table 4, with the increase of cold-start nodes, the performance of all models shows a downward trend. In the cold-start scenario where $M \leq 10$, the AUC of GCNMDA and SCSMDA decreased by up to $2.6\%$ and $2.8\%$ respectively, which verifies the significant challenge to model learning under sparse association data. Under this strict condition, AEPMA demonstrates a significant advantage. Its AUC still remains at $0.966$, which is $3.8\%$ higher than that of the next best-performing model. This result indicates that AEPMA can effectively capture the complex associations in biological networks by autoevolutionary heterogeneous graphs and dynamically focusing on important neighbors.

**Table 4:** Comparison of AUC and AUPRC values of different models on PMDHAN datasets

Method	P$\leq $1 (719)		P$\leq $2 (2081)		M$\leq $1 (27)		M$\leq $5 (115)		M$\leq $10 (250)	
	AUC	AUPRC	AUC	AUPRC	AUC	AUPRC	AUC	AUPRC	AUC	AUPRC
GCNMDA	0.950	0.953	0.948	0.951	0.953	0.953	0.944	0.947	0.928	0.937
MMGCN	0.938	0.939	0.936	0.937	0.941	0.942	0.932	0.933	0.922	0.925
MKGCN	0.910	0.920	0.908	0.915	0.910	0.921	0.903	0.913	0.893	0.901
SCSMDA	0.944	0.941	0.943	0.938	0.944	0.939	0.940	0.937	0.924	0.923
AEPMA	**0.984**	**0.984**	**0.981**	**0.982**	**0.987**	**0.987**	**0.978**	**0.978**	**0.966**	**0.967**

Bold text in the table indicates the highest value in each column.

### Analysis of the generalization of the model

To verify the generalization ability of the AEPMA model, we compared it with other methods on three independent datasets: MDAD [31], aBiofilm [32], and Virus [33]. The MDAD database contains 5505 clinically or experimentally validated microbe-drug associations involving 1388 drugs and 174 microbes. After removing redundant information, we obtain 2470 associations between 1373 drugs and 173 microbes. The aBiofilm database includes information on over 140 microbes and 1720 unique antimicrobial drugs. After repeated screening, we have 2884 MADs covering 1720 drugs and 140 microbes. The Virus database comprises associations between 175 drugs and 95 human viruses, with 933 drug-virus associations that have been clinically or experimentally validated. Detailed information on these three datasets is presented in Table 6.

**Table 5:** Comparison of AUC and AUPRC values of different models on three datasets

Method	MDAD		aBiofilm		Virus	
	AUC	AUPRC	AUC	AUPRC	AUC	AUPRC
GCNMDA	0.930$\,\pm\, $0.0055	0.919$\,\pm\, $0.0094	0.941$\,\pm\, $0.0023	0.929$\,\pm\, $0.0044	0.833$\,\pm\, $0.0063	0.805$\,\pm\, $0.0088
MMGCN	0.894$\,\pm\, $0.0046	0.903$\,\pm\, $0.0073	0.904$\,\pm\, $0.0035	0.910$\,\pm\, $0.0041	0.795$\,\pm\, $0.0074	0.784$\,\pm\, $0.0086
MKGCN	0.981$\,\pm\, $0.0037	0.940$\,\pm\, $0.0062	0.983$\,\pm\, $0.0035	0.952$\,\pm\, $0.0036	0.954$\,\pm\, $0.0063	0.904$\,\pm\, $0.0075
Graph2MDA	0.956$\,\pm\, $0.0039	0.938$\,\pm\, $0.0098	0.927$\,\pm\, $0.0029	0.948$\,\pm\, $0.0034	0.771$\,\pm\, $0.0069	0.795$\,\pm\, $0.0072
SCSMDA	0.957$\,\pm\, $0.0020	0.946$\,\pm\, $0.0033	0.965$\,\pm\, $0.0026	0.945$\,\pm\, $0.0037	0.883$\,\pm\, $0.0064	0.864$\,\pm\, $0.0096
AEPMA	**0.981$\,\pm\, $0.0035**	**0.982$\,\pm\, $0.041**	**0.987$\,\pm\, $0.0033**	**0.988$\,\pm\, $0.0039**	**0.971$\,\pm\, $0.0078**	**0.966$\,\pm\, $0.0081**

Bold text in the table indicates the highest value in each column.

**Table 6:** Information on different drug–microbe association datasets

Dataset	Drugs	Microbes	Associations
MDAD	1373	173	2470
aBiofilm	1720	140	2884
Virus	175	95	933

Based on the above benchmark datasets, we quantified the model performance in terms of AUC/AUPRC metrics through five-fold cross-validation, as shown in Table 5. The experimental results show that AEPMA exhibits excellent stability in cross-domain testing: the AUC reaches $0.981$ on the MDAD dataset, demonstrating its fundamental performance advantage; the AUC reaches $0.987$ in the biofilm-specific aBiofilm scenario, which verifies the model’s ability to parse the complex microbial community; Particularly, it maintains a high AUC value of $0.971$ on the small-sample Virus dataset, highlighting its robustness in data-scarce scenarios. The model’s cross-domain generalization advantage comes from its ability to capture domain-specific associations and adapt to data distribution differences.

### Prediction of potential PMAs

**Figure 7:**
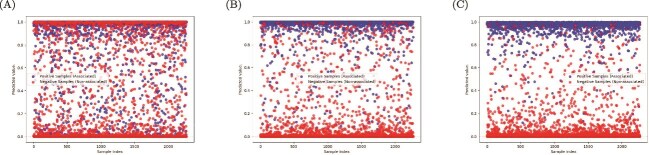
Visualization of PMA’s predicted score across different epochs in PMDHAN. (A) PMA scores visualization at epoch 0. (B) PMA scores visualization at epoch 50. (C) PMA scores visualization at epoch 100.

Based on the constructed heterogeneous network and the AEPMA model, we predict potential PMA. Using a heterogeneous network that includes peptides, microbes, and diseases, we train the AEPMA model with an equal number of positive and negative samples to predict PMA. The trained AEPMA model is then used to predict peptide-microbe relationships in the test set, and the predicted PMA scores are visualized in Fig. 7 using the matplotlib tool. From Fig. 7, it can be observed that the model’s ability to distinguish between positive and negative samples gradually improves. The majority of positive samples have prediction scores concentrated near 1, while the prediction scores of negative samples are mainly clustered around 0. Additionally, the prediction scores of potential negative samples are close to 1, and a small number of positive and negative samples, affected by noise and errors, are distributed in the range of 0.2 to 0.8. Overall, the results indicate that the model can effectively differentiate between positive and negative PMAs.

Additionally, to more concretely evaluate the model’s practicality, we select *Staphylococcus aureus* (*S. aureus*) and *Escherichia coli* (*E. coli*) as experimental subjects. These two bacteria are common pathogens associated with skin and bloodstream infections, as well as gastrointestinal and respiratory tract infections, respectively. We input the features of these two bacteria into the model and obtain the top 10 potential PMA predictions. As shown in Table 7, it lists the peptide sequences, $\log \text{MIC}\ (\mu \mathrm{M})$ values, and activity labels. The label is either “Yes” or “No,” where “Yes” indicates that the peptide has antibacterial activity against the target strain, while “No” indicates that it does not possess such activity. It is worth noting that the activity prediction of each peptide was completed through the AMPActiPred platform (https://awi.cuhk.edu.cn/∼AMPActiPred/) [34], confirming the accuracy of the model’s predictions. We also applied the comparative models to the PMDHAN dataset and compared the results of the top 5 predicted peptides for each model against *E. coli* and *S. aureus*. The experimental results are detailed in the Supplementary Material S5.

**Table 7:** The prediction and validation of novel (potential) PMAs

Microbes	Peptides	$\log \text{MIC}\ (\mu \mathrm{M})$	Label
*E. coli*	AQAAHQAAHAAHQF	1.566	Yes
*E. coli*	AKAKCRKGRAAKRK	0.898	Yes
	KLKGVCRIKGRLKRLAAR		
*E. coli*	KLKKLLKRWRRWWR	0.829	Yes
*E. coli*	ARARCRRGRAARRR	1.300	Yes
	RLRGVCRIRGRLRRLAAR		
*E. coli*	IVRVAVALRRIR	-	No
*S. aureus*	WLRRIKAWLRRKRK	0.820	Yes
*S. aureus*	FFRKVLKLIRKIWR	0.605	Yes
*S. aureus*	DDDKTEEEDDKENE	-	No
	TTKVVE		
*S. aureus*	GLRRALLRLLRSLR	0.964	Yes
	RLLLRAC		
*S. aureus*	RKPRGLEPRRRKVK	-	No
	TTVVYGRRRSKSRERRAP		
	TPQR		

“-” indicates that the peptide does not possess such activity.

We further select a representative peptide from the top-ranked prediction results for detailed analysis. Peptides KLKKLKKLCCLKL are effective against both *Pseudomonas aeruginosa* and Bacillus subtilis, indicating strong membrane interaction and broad-spectrum potential [35]. Given the structural similarity between *B. subtilis* and *S. aureus*, and the amphipathic nature of the peptide, the peptide also likely to exhibit activity against *S. aureus* [36]. To validate the prediction accuracy, peptide-membrane docking and Molecular Dynamics simulations are conducted. Detailed information is provided in Supplementary Material S6.

## Discussion and conclusion

Despite recent advancements in biomedical research and technology, the prediction of PMA remains a challenging task that necessitates the effective learning of information from large-scale heterogeneous networks formed by peptides and microbes. This study proposed a deep learning framework, AEPMA, based on autoevolutionary heterogeneous graphs, to deeply explore the potential mechanisms of PMA. The autoevolutionary heterogeneous graph effectively captures the temporal evolution characteristics of PMDHANs by dynamically embedding the heterogeneous network at different timestamps. Specifically, the method learns the features of network nodes at each time point and updates these features using information propagation patterns from historical timestamps, thereby constructing a series of time-dependent network embeddings. Notably, the information propagation patterns are not fixed but are automatically selected from all possible information aggregation modes, allowing the autoevolutionary heterogeneous graph to flexibly adapt to different network structures and dynamic changes. For example, in the PMDHAN, the information propagation paths of different edge types form a rich set of aggregation modes, encompassing biologically relevant interactions such as “microbe$\rightarrow $peptide,” “peptide$\rightarrow $microbe,” “microbe$\rightarrow $disease,” and so on. Additionally, the model enables information propagation across the time dimension, where features from previous timestamps can influence the node features at the current timestamp, or the current timestamp’s features can remain entirely independent of historical information, enhancing the model’s flexibility and adaptability. More importantly, black-box models lack interpretability, whereas this method demonstrates good interpretability in uncovering the principles of biological interactions.

The outstanding generalization performance of AEPMA across datasets such as MDAD, aBiofilm, and Virus underscores its effectiveness in various contexts. This success can be attributed to its autoevolutionary mechanism, which dynamically adjusts information propagation paths to accommodate the unique characteristics and data distributions of each dataset. This adaptability enables AEPMA to effectively capture domain-specific associations and mitigate data variability across diverse biological scenarios.

Current models rely on the established heterogeneous network structure and the relationships among its nodes for learning and prediction. Therefore, when new peptides or microbes are introduced, it is often necessary to reconstruct the network to incorporate these new nodes and edges. This requires redesigning the node feature representations and edge weight computation mechanisms, as well as retraining the model parameters to accommodate the altered feature distribution. Such a process can be computationally intensive, inefficient, and lacks adaptability to previously unseen data. Future research could prioritize the development of more generalizable node representation methods, such as leveraging embeddings generated by pre-trained models, to enhance the model’s capacity to effectively represent and generalize to novel data.

**Key Points**

- The prediction of peptide-microbe associations (PMAs) is essential in medicine field, and one of the fundamental challenges is how to effectively learn the embedding of nodes and edges in heterogeneous network. Here, we propose an autoevolutionary heterogeneous graph-based PMA prediction model (AEPMA), which serves as an adaptive and efficient method for PMA prediction in a heterogeneous network.
- AEPMA proposes a autoevolutionary heterogeneous graph modeling strategy that dynamically embeds and adaptively selects information aggregation modes, enabling flexible capture of the temporal evolution characteristics of the PMDHAN, which is key to achieving optimal results.
- The effectiveness of the proposed AEPMA is verified on four benchmark datasets. Experimental results demonstrate that our approach outperforms five state-of-the-art methods in predicting PMA. It also provides crucial meta-paths for PMA prediction, providing valuable insights into PMA-related research and enhances interpretability compared to prior black-box deep learning models.

## Supplementary Material

Supplementary_bbaf334

## Data Availability

The datasets used in this study are publicly available and can be accessed at https://github.com/ahu-bioinf-lab/AEPMA.
