# Supplementary material for: AEPMA: peptide–microbe association prediction based on autoevolutionary heterogeneous graph learning
Source: Brief Bioinform. 2025 Jul 10;26(4):bbaf334. doi: 10.1093/bib/bbaf334 (PMC12240738; doi:10.1093/bib/bbaf334)
Supplement: Supplementary_bbaf334 [file supplementary_bbaf334.docx]

**Supplementary Information:**

**AEPMA: peptide-microbe association prediction based on autoevolutionary heterogeneous graph learning**

Zhiyang Hu,^1,2^ Linqiang Pan,^1^ Daijun Zhang,^3^ Yannan Bin^3,*^ and Yansen Su^2,*^

^1^School of Artificial Intelligence and Automation, Huazhong University of Science and Technology, Wuhan, 430070, China, ^2^Information Materials and Intelligent Sensing Laboratory of Anhui Province, Anhui University, Hefei, 230601, China and ^3^Institute of Artificial Intelligence, Hefei Comprehensive National Science Center, 5089 Wangjiang West Road, Hefei, 230088, China

Email: [ynbin@ahu.edu.cn](mailto:ynbin@ahu.edu.cn); [suyansen@ahu.edu.cn](mailto:suyansen@ahu.edu.cn)

**S1 Peptide similarity calculation**

Peptides consist of a long sequence of amino acids and play various roles in organisms. Therefore, by employing the Smith-Waterman algorithm to calculate similarity scores between peptides, local fragments can be aligned on two sequences, revealing to some extent the homology between the two sequences. Subsequently, the similarity scores can be normalized to the range [0,1] using the normalization method proposed in previous work. The normalization formula is as follows:

$score\left(p_{i},p_{j}\right)=S\left(p_{i},p_{j}\right)/\sqrt{S\left(p_{i},p_{i}\right)}\sqrt{S\left(p_{j},p_{j}\right)},$ (1)

where $S\left(p_{i},p_{j}\right)$ is the similarity score obtained by the Smith-Waterman algorithm for $p_{i}$ and $p_{j}$. $S\left(p_{i},p_{i}\right)$ and $S\left(p_{j},p_{j}\right)$ are similarity scores between themselves.

**S2 Microbe similarity calculation and disease similarity calculation**

Considering that two similar diseases are associated with two similar microbes each, there are similar patterns of association and non-association between diseases and microbes^1^. Based on the known microbe-disease associations, the Gaussian interaction profile kernel similarity of diseases (*GD*) and microbes (*GM*) is constructed to represent the similarity between diseases and microbes. Firstly, by observing whether there exist known associations between diseases and each microbe (i.e., the *i-th* row of the adjacency matrix *A*), a binary vector *A*(*d_i_*) is defined to represent the association features of disease *d_i_*. Microbes follow a similar process. The Gaussian interaction profile kernel similarity between diseases *d_i_* and *d_j_*, as well as between microbes *m_i_* and *m_j_*, is calculated as follows:

$GD\left(d_{i},d_{j}\right)=\exp\left(-\eta_{d}\left\|A\left(d_{i}\right)-A\left(d_{j}\right)\right\|^{2}\right),$ (2)

$GM\left(m_{i},m_{j}\right)=\exp\left(-\eta_{m}\left\|A\left(m_{i}\right)-A\left(m_{j}\right)\right\|^{2}\right),$ (3)

where $\eta_{d}$ and $\eta_{m}$ represent the normalized kernel bandwidths, and they are defined as follows.

$\eta_{d}=\eta_{d}^{'}/\frac{1}{nd}\sum_{i=1}^{nd} \left\|A\left(d_{i}\right)\right\|,$ (4)

$\eta_{m}=\eta_{m}^{'}/\frac{1}{nm}\sum_{i=1}^{nm} \left\|A\left(m_{i}\right)\right\|,$ (5)

where $\eta_{d}^{'}$ and $\eta_{m}^{'}$ are the original bandwidths and both are set to 1.

**S3 Experimental Evaluation of Models Runtime Performance on CPU and GPU**

To systematically validate the trade-off between model performance gain and computational overhead, we quantitatively evaluated the runtime of each model on both CPU and GPU computing platforms. Specifically, To ensure the scientific validity and fairness of the experimental results, this study employed a 5-fold cross-validation method to rigorously evaluate the running times of the AEPMA model in comparison to various baseline models. It is important to note that both the GCNMDA and Graph2MDA models are implemented using TensorFlow version 1, which necessitates an operating environment compatible with CUDA 10 or earlier. However, graphics cards from the 30 series and later do not support CUDA versions below 11. Consequently, to facilitate a uniform comparison across all models, the experiments were conducted in a CPU environment. The experimental set up utilized an Intel Core i7-12700 processor with 32GB of memory, ensuring consistency in performance evaluation.

**Table S1**. Performance Comparison of the Running Times of Various Models in the CPU Environment

| Model | GCNMDA | Graph2MDA | SCSMDA | MMGCN | MKGCN | AEPMA |
| --- | --- | --- | --- | --- | --- | --- |
| Running time (minutes) | 25.127 | 59.707 | 139.993 | 945.767 | 270.876 | 56.902 |

For the models SCSMDA, MMGCN, MKGCN, and AEPMA, which are implemented using the PyTorch framework, it is important to consider the memory requirements for optimal performance in a GPU environment. Specifically, the MMGCN model requires more than 30GB of video memory, while the MKGCN model requires more than 40GB. To address these requirements and ensure uniformity in testing conditions, this study utilized an Nvidia A6000 graphics card with 48GB of video memory, which is compatible with CUDA versions above 10. This approach was adopted to maintain consistent experimental conditions across all models and to enhance the reliability of the obtained results.

**Table S2**. Performance Comparison of the Running Times of Various Models in the GPU Environment

| Model | SCSMDA | MMGCN | MKGCN | AEPMA |
| --- | --- | --- | --- | --- |
| Running time (minutes) | 34.112 | 13.214 | 76.551 | 2.715 |

The experimental data indicate that in a CPU environment, the running time of the AEPMA model is 56.902 minutes, positioning it second among all models evaluated. In comparison, the GCNMDA model ranks first. The AEPMA model enhances data interaction by constructing both a microbe-disease association network and a disease similarity network, thereby significantly expanding the dimensionality of the data. Additionally, it innovatively incorporates an autoevolution mechanism to facilitate dynamic optimization of the information propagation path. While these architectural designs and algorithmic innovations have significantly improved the prediction accuracy of the model, they have also increased computational complexity, leading to an extension of the running time. However, the performance advantages of the AEPMA model allow it to maintain a strong competitive edge in the overall evaluation. In a GPU environment, the AEPMA model demonstrates excellent running efficiency, with a running time significantly lower than that of other models, achieving a time savings of 10.5 minutes compared to the model with the second-best performance. It is worth noting that in terms of key evaluation indicators, the AEPMA model demonstrates an average improvement of 2.4% and 2.5% in the AUC and AUPRC indicators, respectively, relative to the model with the second-best performance. These results underscore that the AEPMA model excels not only in running efficiency but also achieves substantial advancements in model performance, thereby showing excellent comprehensive cost-effectiveness.

**S4 Experimental Evaluation of Model Performance Impact via Heterogeneous Network Edge Type Ablation**

Since the proposed method relies on a heterogeneous network structure with multiple edge types, it is necessary to analyze how different edge types influence model performance. To this end, we introduced additional evaluation metrics and conducted further experiments using five-fold cross-validation. Table S3 presents the AUC, AUPRC, and other metrics obtained after removing different edge types. As Table S3 shown, removing the "microbe–peptide" (MP) and "peptide–microbe" (PM) edges resulted in a 9% decrease in Recall and a 5% decrease in F1 Score, respectively, compared to the full-model scenario with all relations retained. Recall measures the model's ability to identify true associations—higher values indicate fewer missed key interactions, which is critical for discovering antimicrobial peptides targeting specific microbes. The F1 Score balances precision and recall, making it particularly suitable for tasks with a high proportion of negative samples. These results highlight the critical role of MP and PM edges in the information propagation process.

**Table S3**. Comparison of Evaluation Indicator Values After Excluding Different Edge Relationships

| Excluded Edges | AUC | AUPRC | ACC | Precision | Recall | F1 Score |
| --- | --- | --- | --- | --- | --- | --- |
| MP | 0.969 | 0.967 | 0.906 | 0.937 | 0.872 | 0.903 |
| PM | 0.968 | 0.967 | 0.904 | 0.935 | 0.869 | 0.901 |
| DM | 0.985 | 0.985 | 0.936 | 0.951 | 0.930 | 0.941 |
| MD | 0.978 | 0.977 | 0.926 | 0.934 | 0.917 | 0.925 |
| PP | 0.983 | 0.984 | 0.936 | 0.939 | 0.929 | 0.935 |
| MM | 0.984 | 0.984 | 0.937 | 0.934 | 0.941 | 0.937 |
| DD | 0.981 | 0.982 | 0.920 | 0.922 | 0.889 | 0.906 |
| Keep all | **0.989** | **0.989** | **0.942** | **0.952** | **0.961** | **0.956** |

**S5 Experimental Evaluation of Model Performance Impact via Heterogeneous Network Edge Type Ablation**

To evaluate the reliability of the prediction results from each model, we applied the comparative models to the PMDHAN dataset and compared the success rates of the top 5 predicted peptides against Escherichia coli (*E. coli*) and Staphylococcus aureus (*S. aureus*) for each model. As shown in Table S4, the sequences, log MIC (μM) values, and activity labels of the top 5 anti- *E. coli* and anti- *S. aureus* peptides predicted by each model are listed. The label is either “Yes” or “No”, where “Yes” indicates that the peptide has antibacterial activity against the target strain, while “No” indicates that it does not possess such activity. Notably, the activity prediction for each peptide was performed using the AMPActiPred platform (<https://awi.cuhk.edu.cn/~AMPActiPred/>). As shown in Table S4, AEPMA achieved activity prediction success rates of 80% and 60% against *E. coli* and *S. aureus*, respectively. Although SCSMDA also showed an 80% success rate against *E. coli*, its success rate against *S. aureus* was only 20%. While both Graph2MDA and MKGCN achieved a 60% success rate against *S. aureus*, their success rates against *E. coli* were 0%. These results indicate that the AEPMA model has higher prediction reliability.

**Table S4**. The activity against *Escherichia coli* and *Staphylococcus aureus* of various peptides predicted using different methods

|  | Model | Anti-*E. coli* active peptide | log MIC (μM) | Label | Anti-*S. aureus* active peptide | log MIC (μM) | Label |
| --- | --- | --- | --- | --- | --- | --- | --- |
|  | GCNMDA | FAKLFAKLAKKFAL | 0.834 | Yes | KKKKAAFLKLWLKLFAA | - | No |
|  |  | YKLLKWLLLKLKALLEKL | - | No | PMNAIKLLCRVHKKIAISV | 1.466 | Yes |
|  |  | WKLFLKAVKKLL | 0.683 | Yes | ILPAKAPAAPARR | - | No |
|  |  | SPKKTKPVKPKKVA | - | No | KWKSFIKNLEKVLKKGPILANLVSIV | - | No |
|  |  | RKFLRRRGEVAHFSQKSLGLYCWWW | 1.074 | Yes | GLLFKELQKLIRYQIFIGK | - | No |
|  | Success rate | 60% | | | 20% | | |
|  | MMGCN | GACAARCRLSSRPRLCHR | 1.199 | Yes | LAHQKPFIRKSYKCLHKRCR | - | No |
|  |  | ILSYLWNGIKSIF | - | No | FIKIFHHIFKGNPKFSIIK | - | No |
|  |  | MNKIFRVIFSKILGRLIVT | 1.121 | Yes | YQWQRRMRKLGAPSIT | - | No |
|  |  | MGKLAIKAGKIIGGGIASALGWAAGEKAVGK | 0.906 | Yes | GACAARCRLSSRPRLCHR | - | No |
|  |  | ILPSKSPSSPSRR | - | No | MNKIFRVIFSKILGRLIVT | 1.111 | Yes |
|  | Success rate | 60% | | | 20% | | |
|  | MKGCN | INWKKIKSIIKAAMN | - | No | SLFSLIKAGAKFLGKNLLKQGACYAACKASKQC | 1.052 | Yes |
|  |  | FXLXAXIXVXLXAXIXL | - | No | GIMSIVKDVAKNAAKEAAKGALSTLSCKLAKTC | 1.369 | Yes |
|  |  | SPKKTKPVKPKKVA | - | No | LRPLLRPLLRPLLRPLLRPLLRPLLRPLLRPL | - | No |
|  |  | QAGSNKGASQKGMS | - | No | AIKIRKLFKKLLR | 0.918 | Yes |
|  |  | GFMDTAKNVAKNVAKNVAVTLLDKLRCKVTGGC | - | No | CNPLNGADRRTDSFPRFTVI | - | No |
|  | Success rate | 0% | | | 60% | | |
| Graph2MDA | | ADSGEGDFLAEGGGVR | - | No | VVFKLASKVVPSVYCTITKK | - | No |
|  |  | VVFKLASKVVPSVYCTITKK | - | No | AWLDKLKSLGKVVGKVALGVAQNYLNPQQ | 1.357 | Yes |
|  |  | GLLSFLPKVIGVIGHLIHPPS | - | No | QSHISLCRWCCNCCKANKGCGFCCKF | 0.772 | Yes |
|  |  | GIGDILKNLAKAAGKAALHAVGESL | - | No | GLWTTIKEGLKKFSLGVLDKIRCKIAGGC | 1.425 | Yes |
|  |  | LFGLIPSLIGGLVSAFK | - | No | RKPRTTKPKPAPKQEPATEE | - | No |
| Success rate | | 0% | | | 60% | | |
|  | SCSMDA | GLMSTLKDFGKTAAKEIAQSLLSTASCKLAKTC | 1.341 | Yes | LKKKKLLKCKLLLKCLKL | - | No |
|  |  | GFMDTAKNVAKNVAKNVAVTLLDKLRCKVTGGC | 1.328 | Yes | SVIGKIASKVVPSVYCAISK | - | No |
|  |  | GLVDVLGKVGGLIKKLLP | 1.554 | Yes | RKSREWRSKKTQPRRPR | - | No |
|  |  | LKRLYKRLAKLIKRLYRYLKKPVR | 0.514 | Yes | AVAALLRAASRAAAA | 1.396 | Yes |
|  |  | GFGSKPLDSFGLNFF | - | No | RHRHRHRHRHRH | - | No |
|  | Success rate | 80% | | | 20% | | |
|  | AEPMA | AQAAHQAAHAAHQF | 1.566 | Yes | WLRRIKAWLRRKRK | 0.820 | Yes |
|  |  | AKAKCRKGRAAKRKKLKGVCRIKGRLKRLAAR | 0.898 | Yes | FFRKVLKLIRKIWR | 0.605 | Yes |
|  |  | KLKKLLKRWRRWWR | 0.829 | Yes | DDDKTEEEDDKENETTKVVE | - | No |
|  |  | ARARCRRGRAARRRRLRGVCRIRGRLRRLAAR | 1.300 | Yes | GLRRALLRLLRSLRRLLLRAC | 0.964 | Yes |
|  |  | IVRVAVALRRIR | - | No | RKPRGLEPRRRKVKTTVVYGRRRSKSRERRAPTPQR | - | No |
|  | Success rate | 80% | | | 60% | | |

**S6 Case analysis**

To validate the accuracy of the prediction results, this study employs peptide-membrane docking (PMD) and Molecular Dynamics (MD) simulations to analyze the interaction between the antimicrobial peptide KLKKLKKLCCLKL and the membrane of *S. aureus*. First, the three-dimensional structure of the peptide is constructed using PEP-FOLD3^3^. Based on previously reported findings^2^, the lipid composition ratio of Gram-positive bacterial membranes is determined, and the peptide–membrane complex system is built using CHARMM-GUI^4^. The simulation system utilizes the CHARMM36 force field and TIP3P water model, and is subjected to energy minimization, equilibration, and a 500 ns production simulation at 310 K using GROMACS^5^. System coordinates are recorded every 100 ns during the simulation. Trajectory analysis is conducted using VMD^6^ and built-in GROMACS tools. Figure 1 illustrates that the peptide gradually inserts into the membrane from the surface, indicating strong affinity for the bacterial membrane. Figure 2 shows the time evolution of key structural parameters of KLKKLKKLCCLKL, including root-mean-square deviation (RMSD), root-mean-square fluctuation (RMSF), and radius of gyration (Rg). Although RMSD fluctuates, it remains relatively low overall, consistent with the progressive insertion observed in Figure 1. RMSF analysis reveals higher values at the N- and C-termini, indicating greater flexibility. Rg results suggest a relatively compact structural conformation, implying that the peptide remains in a surface-bound or shallowly inserted state with a stable fold and dynamic behavior. Collectively, the PMD and MD simulation results further support the accuracy of AEPMA's prediction of antimicrobial peptide activity.


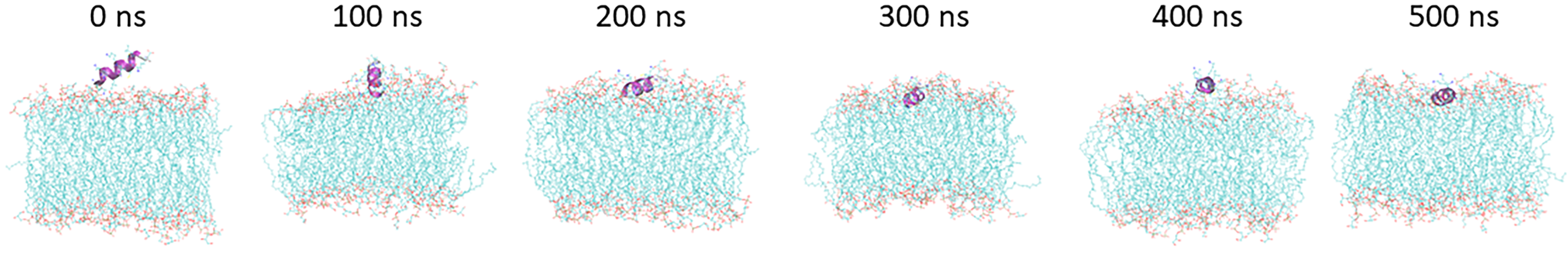


**Figure 1.** VMD visualization of peptide KLKKLKKLCCLKL interacting with the *S. aureus* membrane via PMD. In the snapshots, red spheres and green sticks represent lipid phosphorus membrane.


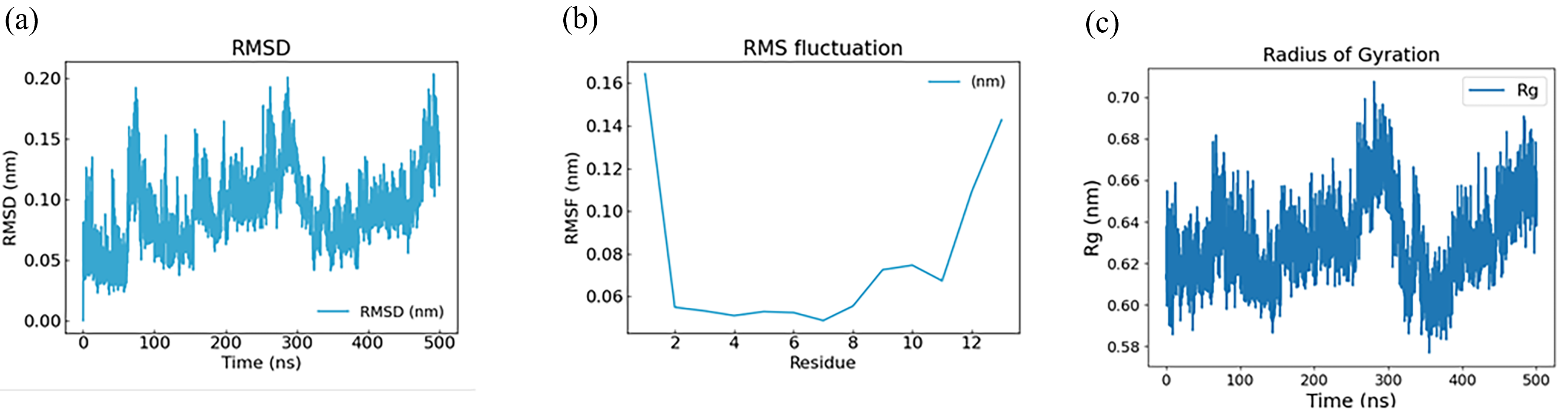


**Figure 2.** The corresponding evolution of key structural parameters for the peptide KLKKLKKLCCLKL interaction with *S. aureus*. (a) RMSD, (b) RMSF, and (c) Rg.

**References**

1. Wei Ma, Lu Zhang, Pan Zeng, Chuanbo Huang, Jianwei Li, Bin Geng, Jichun Yang, Wei Kong, Xuezhong Zhou, and Qinghua Cui. An analysis of human microbe–disease associations. Briefings in bioinformatics, 18(1):85–97, 2017.
2. Hasannejad-Asl B, Heydari S, Azod F, et al. Peptide-membrane docking and molecular dynamic simulation of in silico detected antimicrobial peptides from Portulaca oleracea’s transcriptome[J]. Probiotics and Antimicrobial Proteins, 2024, 16(5): 1501-1515.
3. Cao Z, Shi Z, Tong M, et al. Synergistic Antimicrobial Mechanism of the Ultrashort Antimicrobial Peptide R3W4V with a Tadpole-like Conformation[J]. Journal of Chemical Information and Modeling, 2024, 64(17): 6838-6849.
4. Jo S, Kim T, Iyer V G, et al. CHARMM‐GUI: a web‐based graphical user interface for CHARMM[J]. Journal of computational chemistry, 2008, 29(11): 1859-1865.
5. Abraham M J, Murtola T, Schulz R, et al. GROMACS: High performance molecular simulations through multi-level parallelism from laptops to supercomputers[J]. SoftwareX, 2015, 1: 19-25.
6. Humphrey W, Dalke A, Schulten K. VMD: visual molecular dynamics[J]. Journal of molecular graphics, 1996, 14(1): 33-38.
